# A rare case report of bezold abscess in a 9-month-old baby

**DOI:** 10.1590/0037-8682-0258-2025

**Published:** 2025-09-29

**Authors:** Elif Gozgec, Cemile Altinkaya

**Affiliations:** 1 Ataturk University School of Medicine, Department of Radiology, Erzurum, Turkey.

**FIGURE 1:**
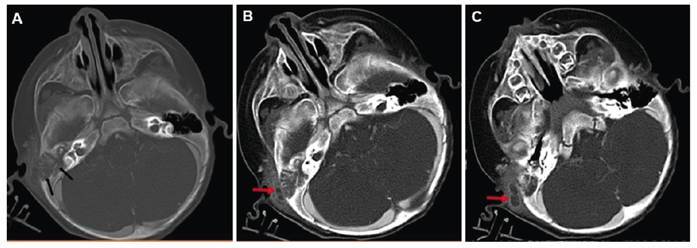
Axial section contrast-enhanced temporal computed tomography images show mastoid cell destruction and diffuse density enhancement (arrows) in the bone window **(A)** and peripherally contrasted abscess localization extending from the preauricular area to the sternocleidomastoid muscle (arrows) in the soft tissue window **(B and C).**

A 9-month-old male infant presented with fever, ear pain, ear discharge, and irritability. Physical examination revealed asymmetric swelling, redness, and elevated temperature in the right preauricular region compared with the left, with evidence of fluctuation. An otoscopic examination revealed redness, effusion, and thickening of the right tympanic membrane. Laboratory tests indicated a white blood cell count of 13,000/μL, with a high percentage of neutrophils. Contrast-enhanced computed tomography (CT) of the temporal region revealed destruction of the right mastoid bone and a peripheral contrast-enhanced hypodense collection extending from the mastoid to the middle ear cavity, right sternocleidomastoid (SCM) muscle, and preauricular subcutaneous tissue (Figure 1). A Bezold abscess was suspected. *Streptococcus pneumoniae* was identified as the causative agent from percutaneous abscess drainage. Ceftazidime and vancomycin treatment was initiated. After 1 week, the patient showed improvement in symptoms, and the white blood cell count decreased to 8,000/μL, allowing for discharge.

Bezold’s abscess is a rare type of deep neck abscess that reaches the SCM through erosion of the mastoid cortex. Since 1967, fewer than 100 cases have been reported, with only four children aged ≤5 years^1^. In the advanced stages, the infection can extend to the infratemporal fossa and carotid sheath, potentially leading to internal jugular vein thrombosis. Contrast-enhanced CT scanning is essential for diagnosis, determination of the extent of infection, and evaluation of the response to treatment. The introduction of antibiotics has significantly reduced the documented incidence of this condition^2^.
